# Ear symptoms in patients with orofacial pain and dysfunction ‐ An explorative study on different TMD symptoms, occlusion and habits

**DOI:** 10.1002/cre2.457

**Published:** 2021-06-01

**Authors:** Christina Mejersjö, Nina Pauli

**Affiliations:** ^1^ Clinic of Orofacial Pain Sahlgrenska Academy at the University of Gothenburg and the Public Dental Health Service Gothenburg Sweden; ^2^ Department of Otorhinolaryngology, Institute of Clinical Sciences Sahlgrenska Academy at the University of Gothenburg, Sahlgrenska University Hospital Gothenburg Sweden

**Keywords:** aural symptoms, chewing side, ear fullness, occlusion, TMD

## Abstract

**Objectives:**

Ear symptoms coincident with TMD symptoms have been noticed for a long time. The aim was to investigate the relationship between reported ear symptoms in TMD patients and different TMD symptoms, dental occlusion, oral parafunction and habits.

**Material and methods:**

Consecutive patients, ≥18 years of age and referred to a specialist clinic for orofacial pain and dysfunction during a three‐month period, were considered for the study. Patients with poor general or psychiatric health were excluded. One hundred thirty‐two patients were included and studied with regard to reported ear symptoms in relation to clinical dysfunction, occlusion, habits and subjective rating of their symptoms. A clinical examination was performed according to RDC/TMD and extended with occlusal factors, parafunctions and habits.

**Results:**

Ear symptoms were reported by 72% of the TMD patients, with ear fullness in 49% as the most frequent symptom. The patients with ear symptoms were significantly older and proportionally more often females. Ear symptoms were significantly correlated to the subjective index, to myalgia (*p* = 0.003), decreased opening capacity (*p* = 0.01), TMJ pain (*p* = 0.02), parafunctions (*p* = 0.007), and some occlusal factor (*p* = 0.018–0.003). Muscle pain on palpation was significantly associated with ear fullness, and changed hearing and sensitivity to sound, on the same side (*p* < 0.005).

**Conclusions:**

Ear symptoms are frequently reported by TMD patients. Concomitant ear symptoms are associated with oral parafunction and muscle pain on palpation on the same side as the ear symptoms.

## 1 INTRODUCTION

Patients with orofacial pain and dysfunction often report coincident ear/aural symptoms, like otalgia, sensation of ear fullness or itching, sensitivity to sound and changed hearing. Such symptoms have been noticed for a long time among patients seeking dental or medical care for temporomandibular dysfunction (TMD) (Keersmaekers et al., 1996; Lam et al., 2001; Mejersjö & Näslund, 2016; Porto De Toledo et al., 2017; Tuz et al., 2003), but little is known about which patients are at risk of being affected.

TMD is a collective term for different types of conditions and symptoms of the stomatognathic system. It is not a diagnosis but includes various diagnoses with different background and pathophysiology (de Leeuw & Klasser, 2013a). Epidemiologically, symptoms of orofacial pain and dysfunction have been reported by about 10% of the population, with the highest frequency among women 20–60 years of age (Lövgren et al., 2016).

In a review of aural symptoms in TMD patients, 43%–96% reported ear fullness and 32%–77% reported otalgia (Porto De Toledo et al., 2017). Of the aural symptoms without an organic ear pathology, ear fullness and otalgia were the most frequent (Maciejewska‐Szaniec et al., 2017; Mejersjö & Näslund, 2016). When patients referred to a specialist clinic due to TMD symptoms with coincident ear symptoms were examined by an otolaryngologist, none had an ear pathology, except for a few patients with impaired hearing (Mejersjö & Näslund, 2016). Tinnitus has usually not been included in ear symptoms without an organic ear pathology, and has not been associated with TMD symptoms in particular (Rubinstein, 1993). Patients with tinnitus but without TMD symptoms are usually not taken care of at the clinic for orofacial pain and dysfunction.

In a study of patients seeking medical attention due to ear pain, 46% of those with referred otalgia were also diagnosed with temporomandibular joint (TMJ) dysfunction (Jaber et al., 2008). In a group of elderly persons in Brazil, ear fullness was reported by 4%–9% (do Carmo et al., 2008), and in a review of ear symptoms in the general population, the prevalence of an ear symptomatology varied from 10% to 31% (Salvetti et al., 2006). The background of the aural symptoms in patients with TMD is not fully know but there are different theories:

Mechanical pressure, exerted by the TMJ on the hearing organ due to their close vicinity, is one explanation (Bush et al., 1999). A deep bite, or hypertonic masticatory muscles, has been considered to result in muscle compression of adjacent structures (Henderson et al., 1992).

Eustachian tube dysfunction, or dysfunction of the tensor tympani muscle, is another possible explanation for the sensation of fullness in the ear (Park et al., 2012). Thirty‐five percent of patients seeking medical attention due to both ear fullness and TMD symptoms were diagnosed with Eustachian tube dysfunction (Peng, 2017), and in another study, 29% of the patients received the same diagnosis (Park et al., 2012).

Common innervation: The ear, the bite muscles, the TMJ and the Eustachian tube with the tensor veli palatini muscles are all innervated by mandibular branches of the trigeminal nerve and have functional neuromuscular interaction. Masticatory muscle hyperactivity could cause referred pain in the ear (M. Kuttila et al., 2002; S. Kuttila et al., 2004) and may lead to increased tonicity of the tensor veli palatini and tensor tympani muscles. A study of patients with bruxism and concomitant ear symptoms supports the theory of association with hyperactivity of the bite muscles (Magalhäes et al., 2018). However, referred pain from a cervical spine degenerative disorder was also found to be associated with otalgia (Jaber et al., 2008).

A common embryologic origin of the TMJ, the lateral pterygoid and the malleus, incus and, in part, the stapes bones of the middle ear (Ramirez et al., 2008; Thilander et al., 1976) could explain some of the associations between the ear and TMD symptoms.

A minor alteration in the connective properties with increased stiffness of the middle ear has been discovered in patients with concomitant TMD and ear symptoms (Riga et al., 2010). Ear symptomatology has also been associated with occlusal factors (Bjorne & Agerberg, 2003; Candido dos Reis et al., 2000). TMJ clicking was significantly associated with otalgia in young Japanese adults (Akhter et al., 2013).

A decrease in ear symptoms has been noticed after treatment of TMD symptoms (Keersmaekers et al., 1996; S. Kuttila et al., 2004). Improvement of both TMD and ear symptoms was found after treatment with occlusal appliance (M. Kuttila et al., 2002; Ramirez et al., 2008) and after occlusal adjustment (Bjorne & Agerberg, 2003). However, reviews on the effect of TMD therapy on aural symptoms have provided insufficient evidence that TMD treatment can change otologic signs and symptoms (Morell, 2018; Stechman‐Neto et al., 2016).

The aim of the study was to investigate the relationship between reported ear symptoms and different dysfunction symptoms, factors of dental occlusion, oral parafunction and habits in patients referred to a TMD clinic, to better understand and treat the ear symptoms.

## 2 MATERIALS AND METHODS

### 2.1 Patients and inclusion criteria

All new patients, referred to the specialist clinic of Orofacial Pain and Dysfunction, the Public Dental Health Service, Gothenburg, and who visited three of the specialists at the clinic during a three‐month period were considered for the study. Inclusion criteria: consecutive patients aged ≥18 years and referred due to various conditions and symptoms of dysfunction. Exclusion criteria: severely impaired general or psychiatric health and patients unable to understand Swedish (four patients). One‐hundred thirty‐two patients were included.

### 2.2 Questionnaires and examination

The patients answered the clinic's validated standard questionnaires (RDC/TMD) (Schiffman et al., 2010), including the SCL 90 index (Symptom CheckList for somatization and depression), and the Characteristic Pain Intensity (CPI) scale (part of the Graded Chronic Pain Scale, GCPS, a questionnaire for grading of pain) (de Leeuw & Klasser, 2013b), and evaluated the degree of the orofacial dysfunction by a subjective 5‐point verbal index (Si) (Magnusson et al., 1995). Patient agreement to participate in the study was obtained and an additional short questionnaire regarding the patient's experience of five different symptoms of the ears was administered shortly before the first appointment at the clinic (Appendix 1 in Data S1). The number of different reported ear symptoms was calculated.

The clinical examination of the patients was performed according to the RDC/TMD (Schiffman et al., 2010). The specialists performing the examination were unaware of the patient's report of ear symptoms. An additional examination (Appendix 2 in Data S1) was performed regarding occlusal interferences (Okeson, 2013), degree of attrition, degree of parafunctions 0–3 (cheek biting, tongue pressure and reported chewing‐gum use ≤4 h/day), facial asymmetry, habits like preferred chewing side (stated by the patient), preferred side for tilting the head and bruxing position. The specialists trained together in advance regarding the examination and recording of the findings.

Occlusal interferences in the retruded contact position (RCP) and mediotrusive contacts were recorded, as well as any lateral slide from the RCP to the intercuspid contact position (ICP), with the direction and size of the slide (Okeson, 2013). Any bruxing position according to facets in the occlusion was noted. The degree of attrition was estimated (Dahl et al., 1993) and the opening of the bite on laterotrusive movement (Abduo & Tennant, 2015) was measured at the canines on both sides.

After the examination, the diagnoses were established and most patients were given more than one diagnosis; for example, myalgia could be combined with the diagnosis of tension headache, parafunction or disc displacement. The dominant diagnosis was given as the main diagnosis. The patients then proceeded to individual treatment at the clinic. The patients' answers to the questionnaires, the reported degree of symptoms, clinical findings and diagnoses were later collected from the patient records. The study was approved by the local ethical review board in Gothenburg, reg. no. 457‐18.

### 2.3 Statistics

The patients were grouped according to “any report of ear symptoms” or “without ear symptoms,” and the groups were compared. Among patients with ear symptoms, associations with reported TMD symptoms and clinical findings were analyzed. When the ear symptoms were bilateral, the reported dominant side, if any, was used for the analysis.

The SPSS statistics software, version 22, was used for the data registration and statistical analysis. The department of Health Metrics at the Sahlgrenska Academy, University of Gothenburg, assisted with the statistical analyses. The statistical methods used were the t‐test, the Chi_2,_ and Fisher's exact tests. A few correlations were analyzed by logistic regression, adjusted for gender and age. A statistical significance level *p* < 0.05 is given.

## 3 RESULTS

### 3.1 Ear symptoms and patient characteristics

The study comprised 132 referred patients, age 18–81 years, mean 46.5 years, 70% of whom were female. Ear symptoms were reported by 72% (*n* = 95), 53% in one ear, 18% in both ears. A sensation of fullness in the ear was the most frequent symptom, reported by 49% of the patients. The frequencies of reported ear symptoms are shown in Table 1. Before referral to the orofacial pain clinic, 39% of the patients had sought medical attention because of their ear symptoms.

**TABLE 1:** Frequency of different reported ear symptoms and side of symptoms in 132 TMD patients

Reported symptom	(%)	Right side	Left side	Bilateral
Ear fullness	49	21	17	11
Otalgia	33	18	9	6
Changed hearing	28	11	9	8
Sensitivity to sound	26	8	5	12
Itching of ear	24	6	4	14

When comparing the groups of patients with and without aural symptoms, those without aural symptoms were younger and proportionally more often males (Table 2). Females reported ear symptoms more often than males (*p* = 0.005) and had a larger number of ear symptoms (*p* < 0.05), while attrition was significantly more frequent in males (*p* = 0.005). Different ear symptoms were often combined, like ear fullness with otalgia and changed hearing.

**TABLE 2:** Distribution of age and gender among TMD patients with and without ear symptoms, *n* = 132 (n.s., not significant)

TMD patients	With ear symptoms	Without ear symptoms	
Age, years			
Mean	48.1	42.6	n.s. (*p* = 0.088)
Range	(18–81)	(19–71)	
Gender, %			
Females	76.8	54.1	
Males	23.2	45.9	*p* < 0.001

### 3.2 TMD diagnoses, reported degree of symptoms

The main diagnosis and additional diagnosis, if any, for the patients with and without reported aural symptoms are shown in Table 3. Myalgia was significantly more frequent among patients with aural symptoms (*p* = 0.003). The number of different reported ear symptoms was correlated with the main diagnosis of myalgia (*p* = 0.005).

**TABLE 3:** Frequency of main and additional, if any, TMD diagnoses of 132 patients referred for orofacial pain and dysfunction, according to reported presence or absence of ear symptoms

TMD patients	Main diagnoses (*n* = 95)	Additional diagnoses (*n* = 84)	Main diagnoses of patients with ear fullness (*n* = 64)
	%	%	%
With ear symptoms			
Myalgia	65.3	13.1	70.3
Arthralgia	11.6	3.6	10.9
DD with reduction	5.3	22.6	4.7
Tension headache	5.3	19.0	6.3
Bruxism, parafunction	5.3	23.8	1.5
Malocclusion, attrition	4.2	14.3	3.1
Sleep apnea	2.1	1.2	1.5
Arthrosis	2.1	4.8	1.5
	(*n* = 37)	(*n* = 32)	
	%	%	
Without ear symptoms			
Myalgia	33.3	25.0	
Arthralgia	8.3	6.3	
DD with reduction	8.3	12.5	
Tension headache	5.6	3.1	
Bruxism, parafunction	13.9	34.4	
Malocclusion, attrition	19.5	12.5	
Sleep apnea	11.1	‐	
Arthrosis	‐	6.3	

*Note*: The main TMD diagnoses of the patients reporting ear fullness, among the 95 patients with aural symptoms, are also shown (DD, disc displacement; *n*, number of patients).

Ear symptom was significantly correlated with the subjective index Si (*p* < 0.01), and with the SCL 90 index, both for somatization (*p* = 0.016) and depression (*p* < 0.05). The CPI value was numerically higher among those with aural symptoms; however, not significantly so. When the diagnoses of myalgia and arthralgia were compared with regard to mean values of Si, SCL 90 and CPI, no significant difference was found.

### 3.3 Clinical signs and correlated aural symptoms (Table 4)

Pain in the bite muscles on palpation was significantly more frequent and of a higher degree among patients with ear symptoms. Of those with muscle pain on mandibular movement, 2/3 had pain only on the same side as the ear symptoms.

**TABLE 4:** Clinical findings among TMD patients with (*n* = 95) and without (*n* = 37) reported ear symptoms

Clinical findings	TMD patients		
	with ear symptoms	without ear symptoms	*p* value
Opening capacity (mean, mm)	44.0	47.5	0.022
Max. laterotrusion right/left (mean, mm)	9.3/9.6	9.8/10.6	
TMJ pain on palpation (%)	40	19	0.016
TMJ pain on movement (%)	16	11	n.s.
Clicking (%)	33	38	n.s.
Crepitation (%)	26	9	0.010
Muscle pain on movement (%)	34	22	n.s.

*Note*: Significant differences between the groups are given with *p* values, n.s., no statistically significant difference.

Ear symptoms were correlated with decreased opening capacity (*p* = 0.01). Pain on palpation of the masseter, the temporal and the neck muscles was significantly associated with ear fullness, with changed hearing and sensitivity to sound on the same side as the ear symptoms (*p* < 0.005), but also on the opposite side of the ear symptoms (*p* < 0.05).

TMJ pain on palpation was correlated with otalgia on the same side (*p* = 0.04), with ear fullness (same side *p* = 0.02, opposite side *p* = 0.01) and with changed hearing on the opposite side (*p* = 0.028). Among patients with pain on palpation of the TMJ, 3/4 felt pain only on the same side as the reported ear symptoms.

TMJ crepitation was correlated with ear symptoms on the opposite side (*p* = 0.002). Only one patient with clinical TMJ crepitation reported no aural symptom. TMJ clicking dominated on the same side as the ear symptoms, but no significant correlation was found with ear symptoms.

### 3.4 The occlusion

Observed occlusal factors are shown in Table 5 and correlations with ear symptoms in Tables 6 and 7. The mean values of the vertical overbite were about the same for both groups, with and without ear symptoms. However, of those with a deep bite 83% had ear symptoms. The average estimated vertical opening during lateral excursion was 3.0 mm for those with and 2.7 mm for those without ear symptoms, and an increased opening was correlated with ear fullness on the same side (*p* = 0.024).

**TABLE 5:** Frequency of occlusal factors noticed among TMD patients with (*n* = 95) and without (*n* = 37) ear symptoms

Occlusal factors	Patients (%)	
	with ear symptoms	without ear symptoms
RCP interference	41	53
Right /left side	28/12	22/31
Lateral slide RCP‐ICP	19	16
To the right/to the left	5/15	13/3
Mediotrusion interferences	30	30
Sagittal distance (mm)		
Mean RCP–ICP	0.5	0.6

**TABLE 6:** Correlations between occlusal factors and reported ear symptoms in 132 TMD patients with Fisher's exact test and *p* value for level of significance

Occlusal factor	Ear symptom	*p* value
RCP‐interference	Changed hearing	0.004
Lateral slide RCP ‐ ICP	Ear fullness	0.003
	Changed hearing	0.004
Mediotrusion interference	Changed hearing	0.006
	Sound sensitivity	0.018

**TABLE 7:** Logistic regression analysis of correlation between ear symptom and occlusion and extensive parafunction, respectively, adjusted for age and gender with *p* value for level of significance

Factors correlated to ear symptoms	Adjustment for	
	gender	gender + age
Lateral slide RCP to ICP	*p* = 0.05	*p* = 0.06
Extensive parafunction	*p* = 0.06	*p* = 0.05

### 3.5 Habits

Observed parafunction was significantly correlated with ear symptoms (*p* = 0.007) and with the number of different ear symptoms. Analyses with logistic regression showed significant correlation between extensive parafunction and ear symptom also when adjusted for gender and age (*p* = 0.05) (Table 7). The chewing side preferred by the patient was correlated with ear symptoms on the same side (*p* = 0.04).

Observed bruxing positions showed no significant correlation with ear symptoms, nor was there any association with the degree of attrition, with facial asymmetry or with the preferred side of tilting the head.

### 3.6 Tinnitus

Tinnitus was reported by 31% of the whole group of patients. There was an association between tinnitus and ear symptoms (*p* = 0.017).

## 4 DISCUSSION

Ear symptoms were frequently reported by patients referred to a specialist clinic because of orofacial pain and dysfunction. Ear fullness was the most commonly reported symptoms, affecting almost 50% of the TMD patients, followed by otalgia. Ear symptoms on the right side dominated. The frequency of symptoms in our study is in accordance with that in previous studies (Porto De Toledo et al., 2017).

A large number of aspects are considered in the study of ear symptoms among TMD patients, with the intention to find associated factors for further studies. This is why it is called an explorative study. Associations with different clinical factors, proposed in the literature and occasioned by clinical observations, were studied. However, the large number of variables and tests performed may involve a risk of mass significance.

Even though ear symptoms are common in TMD patients, a differential diagnosis of a more severe disorder must be considered and assessed by a specialist with enough competence. TMD patients with concomitant unilateral auditive symptoms, like hearing disturbance or ear fullness, who do not quickly respond to the conservative treatment of orofacial pain and dysfunction, should be referred to an otolaryngologist to rule out a skull base pathology such as vestibular schwannoma. Likewise, patients with unilateral ear symptoms and persistent pain in the head and neck region must be assessed by an otolaryngologist in order to rule out head and neck neoplasms.

A strong association was found between ear symptoms and muscle pain on palpation, especially on the same side, which could be explained by the common innervation from different branches of the trigeminal nerve. The auriculotemporal branch of the trigeminal nerve, which almost exclusively innervates both the tympanic membrane (Williams, 1999) and the TMJ, may explain aural symptoms like ear fullness due to pain of the masticatory muscles. Our study found a strong association between neck muscle pain on palpation and ear symptoms, perhapes with the same background as spine disorders and ear symptoms (Jaber et al., 2008; S. Kuttila et al., 2004). The ear symptoms could possibly be due to peripheral and central sensitization with changes like lowered nerve thresholds and an enlarged receptive field (de Leeuw & Klasser, 2013c).

A diagnosis of arthralgia was not significantly associated with aural symptoms, while pain on palpation of the TMJ and TMJ pain on movement were. Arthralgia may be due to arthritis but is also caused by overloading of the joint from tension and hyperactivity. Treatment of arthralgia with intra‐articular injection of saline was superior to treatment with corticosteroids, indicating a non‐inflammatory condition (Isacsson et al., 2019). No correlation was found between ear symptoms and TMJ clicking, contrary to the results of a Japanese study (Akhter et al., 2013).

Ear symptoms were associated with the diagnosis of myalgia, with oral parafunction and the preferred side of chewing, which could support an association with tension or hyperactivity as has been reported previously (Magalhäes et al., 2018). The degree of parafunction was estimated from signs in the oral mucosa noted at the clinical examination and was influenced by the patient only regarding the reported amount of chewing‐gum use. A large lateral opening on laterotrusion has sometimes been regarded as favorable, but in this study a greater lateral bite opening on laterotrusion was associated with ear symptoms. This could possibly be due to an orthodontical effect from extended parafunctional biting in the ICP and a deepening of the bite.

Crepitation of the TMJ was correlated with ear symptoms but on the opposite side of the ear symptoms. Arthrosis is clinically identified by joint crepitation, and has a background including loading. The association could possibly be explained by the development of arthrosis in the loaded TMJ opposite to the side of the myalgia from prolonged tension.

The role of occlusal interferences for TMD symptoms overall has been discussed over the years, although regarded as being of minor importance in modern literature (Manfredini et al., 2017). However, in this study, some occlusal interferences were associated with the ear symptoms. It is important to realize that the occlusion is not static but changes according to function and forces, and that interferences may develop from bruxism or prolonged biting. Whether the association between ear symptoms and occlusal interferences is a causal relationship, or if both are consequences of hyperactivity and parafunction, cannot be determined from this study.

TMD patients with ear symptoms reported more subjective symptoms of orofacial pain and dysfunction, and had a higher depression and somatization index compared with the patients without aural symptoms. This could have been influenced by the concomitant ear symptoms. TMD has been found to be strongly related to depression and somatization levels (Manfredini et al., 2010), and the ear symptoms may, to some extent, be a form of somatization. The clinical dysfunction noted was also significantly greater among those with concomitant ear symptoms and has been suggested to represent more severe orofacial pain and dysfunction (Maciejewska‐Szaniec et al., 2017).

A limitation of the study is that the ear symptoms asked after were reported by the patients, and that no medical examination of the patients was performed. In an earlier study (Mejersjö & Näslund, 2016) from the same clinic, the TMD patients with ear symptoms were also examined by an otolaryngologist but, with the exception of some cases with impaired hearing, no pathology of the ears was found. In the present group of TMD patients, 39% reported that they before the examination at the orofacial pain and dysfunction clinic, had had a medical consultation because of their ear symptoms, some had been treated but without a change and some of them had been referred to the TMD specialist.

Another limitation of the study is that the patients only answered with “yes” or “no” to the symptoms asked after, which does not say anything about the frequency or intensity of the ear symptoms. The period of the symptoms asked after was long with difficulties to remember pain (Feine et al., 1998), but a year corresponds to a common waiting time for patients, from referral until they were examined by a specialist for their orofacial pain and dysfunction.

It is important to exclude potentially severe differential diagnoses and clinics of orofacial pain and ear, nose and throat clinics should strive to collaborate closely to provide the best care possible for these patients.

## 5 CONCLUSION

Aural symptoms accompanying TMD symptoms are found in more than half the group of TMD patients. Concomitant ear symptoms were associated with oral parafunction and with muscle pain on palpation on the same side.

## CONFLICT OF INTEREST

The authors state that there is no conflict of interest in connection with this article.

## AUTHOR CONTRIBUTIONS

Christina Mejersjö has initiated the study, and collected the patients' answers to the questionnaires, the reported degree of symptoms, clinical findings and diagnoses noted in the patient records. Nina Pauli and Christina Mejersjö discussed the results and wrote the manuscript, and the statistical analysis was performed in cooperation with the department of Health Metrics at the Sahlgrenska Academy, University of Gothenburg.

## Supporting information


**Data S1.** Supporting Information.

## Data Availability

Data are available at the Clinic of Orofacial Pain, Sahlgrenska Academy at the University of Gothenburg and the Public Dental Health Service, Gothenburg, Sweden.
