# Supplementary material for: Ear symptoms in patients with orofacial pain and dysfunction ‐ An explorative study on different TMD symptoms, occlusion and habits
Source: Clin Exp Dent Res. 2021 Jun 1;7(6):1167–74. doi: 10.1002/cre2.457 (PMC8638311; doi:10.1002/cre2.457)
Supplement: Supplementary file 1 — Data S1. Supporting Information. [file CRE2-7-1167-s001.docx]

**Appendix 1 Questionnaire regarding aural symptom during the last year:** “Mark any ear symptoms that you experienced during the past year concomitant with the jaw and head symptoms for which you have been referred, and if you have had a medical consultation or have been prescribed medication for your ear problems”:

yes side

Pain in the ear

Feeling of fullness or swelling in the ear

Experience of impaired hearing or loss of hearing ­­

Hyper sensitivity to sounds

Itching in the ear

Medical consultation due to the ear symptoms

Prescription of antibiotics due to the ear symptoms

**Appendix 2 The extended clinical examination included:**

- preferred side of chewing

- right or left-handed

- side of tilting the head

- facial asymmetry, shortest side

- the best ear when listening attentively

- RCP interference and side

- lateral slide RCP-ICP towards …. Slide in mm …

- mediotrusion contact/interference and side

- opening on laterotrusion at the canine; towards right …mm, type of contact…

towards left….mm, type of contact…

- any bruxoposition, location
